# Selecting putative drought-tolerance markers in two contrasting soybeans

**DOI:** 10.1038/s41598-022-14334-3

**Published:** 2022-06-27

**Authors:** Laila Toum, Lucia Sandra Perez-Borroto, Andrea Natalia Peña-Malavera, Catalina Luque, Bjorn Welin, Ariel Berenstein, Darío Fernández Do Porto, Adrian Vojnov, Atilio Pedro Castagnaro, Esteban Mariano Pardo

**Affiliations:** 1 Instituto de Tecnología Agroindustrial del Noroeste Argentino, Estación Experimental Agroindustrial Obispo Colombres- Consejo Nacional de Investigaciones Científicas y Técnicas (CONICET), William Cross 3150, Las Talitas, Tucumán Argentina; 2 Plant Breeding, Wageningen University & Research, 6708 PB Wageningen, The Netherlands; 3 Centro de Bioplantas, Universidad de Ciego de Ávila “Máximo Gómez Báez”, Road to Morón 9 ½ Km, Ciego de Ávila, Cuba; 4 Cátedra de Anatomía Vegetal. Facultad de Ciencias Naturales E IML, Universidad Nacional de Tucumán, Miguel Lillo 205, San Miguel de Tucumán, Tucumán Argentina; 5 Laboratorio de Biología Molecular, División Patología, Instituto Multidisciplinario de Investigaciones en Patologías Pediátricas (IMIPP), CONICET-GCBA, C1425EFD Buenos Aires, Argentina; 6 Instituto de Química Biológica (IQUIBICEN), Facultad de Ciencias Exactas y Naturales (FCEyN), Universidad de Buenos Aires, Intendente Guiraldes 2160, Buenos Aires, Argentina; 7 Instituto de Ciencia y Tecnología “Dr. César Milstein”, Fundación Pablo Cassará-Consejo Nacional de Investigaciones Científicas y Técnicas (CONICET), Saladillo 2468, C1440FFX Buenos Aires, Argentina

**Keywords:** Plant breeding, Plant stress responses

## Abstract

Identifying high-yield genotypes under low water availability is essential for soybean climate-smart breeding. However, a major bottleneck lies in phenotyping, particularly in selecting cost-efficient markers associated with stress tolerance and yield stabilization. Here, we conducted in-depth phenotyping experiments in two soybean genotypes with contrasting drought tolerance, MUNASQA (tolerant) and TJ2049 (susceptible), to better understand soybean stress physiology and identify/statistically validate drought-tolerance and yield-stabilization traits as potential breeding markers. Firstly, at the critical reproductive stage (R5), the molecular differences between the genotype’s responses to mild water deficit were explored through massive analysis of cDNA ends (MACE)-transcriptomic and gene ontology. MUNASQA transcriptional profile, compared to TJ2049, revealed significant differences when responding to drought. Next, both genotypes were phenotyped under mild water deficit, imposed in vegetative (V3) and R5 stages, by evaluating 22 stress-response, growth, and water-use markers, which were subsequently correlated between phenological stages and with yield. Several markers showed high consistency, independent of the phenological stage, demonstrating the effectiveness of the phenotyping methodology and its possible use for early selection. Finally, these markers were classified and selected according to their cost-feasibility, statistical weight, and correlation with yield. Here, pubescence, stomatal density, and canopy temperature depression emerged as promising breeding markers for the early selection of drought-tolerant soybeans.

## Introduction

Soybean [*Glycine max* (L.) Merrill] represents one of the most important sources of vegetable oil and protein in the world^1^. Calculation models based on the growing global population and current agricultural production suggest that crop yields, including soybean, must be doubled to provide enough food in 2050^2^. Yield, the principal breeding target for most crop plants, is massively affected by suboptimal growth conditions primarily due to climate factors such as drought and extreme temperatures. In addition, the progressive climate change will reduce water availability for many rainfed crops like soybean, affecting their growth and productivity^3^. Hence, breeding for yield stabilization and drought tolerance in soybean and other crops is essential for sustainable agriculture and food production^4^.

The development of cultivars with improved yield under water deficit has had relatively limited success for several reasons. First, the direct selection for yield improvement under drought is expensive, time-consuming, laborious, and complex due to intrinsic genotype by environmental interactions^5^. Moreover, when determining plant performance under drought conditions, inherent stress susceptibility is often masked by the spillover effects of high yield potential. Consequently, a high-yield variety will often give significant yields during drought, even though the relative yield reduction can be substantial^6^. Instead, analytical approaches that emphasize breeding for yield stabilization through an indirect selection strategy, using morphophysiological or biochemical markers, have gained increasing attention^7,8^. However, the challenge for effectively using targeted breeding approaches lies in developing reliable and reproducible markers. These markers should be (i) strongly related with yield and stress-tolerance traits, where possible, (ii) be non-destructive, (iii) be easily measurable in early phenological stages, and (iv) have a high narrow-sense heritability to facilitate the selection in breeding populations^9^. Therefore, identifying and validating drought-tolerance traits are essential steps to obtaining valuable markers for breeding programs and selecting superior genotypes. Soybean drought tolerance has been evaluated through markers such as water use efficiency (WUE), root morphology and penetrability of hardpan, leaf wilting, excised leaf water loss, and relative water content (RWC) with varying degrees of success^10^.

Advances in next-generation sequencing (NGS) and subsequent evolution in multi-omics technologies have contributed to understanding some underlying mechanisms of response to water deficit in soybean and other crops^11^. Omics studies, however, must be complemented with morphophysiological and biochemical approaches to ensure an integrative perspective of plant adaptation to water scarcity and accurately assess the role of individual traits regarding stress tolerance and yield. Usually, the main issue in such studies is the lack of a well-defined and reliable phenotyping methodology that validates the trait's accuracy^12^. It is safe to say that, currently, phenotyping systems are the major operational bottleneck in plants breeding, limiting the translation of genetic and genomic analysis into stress-tolerant phenotypes.

In previous research^13^, our group phenotyped several soybean genotypes submitted to drought treatments during the reproductive stage in greenhouse and field conditions. Comparative studies were performed in commercial cultivars and the widely studied PI 416937, a reference slow-wilting genotype. Overall, MUNASQA exhibited the lowest yield loss and yield-based Drought Susceptibility Index (DSI) under water scarcity conditions, while TJ2049 showed the opposite behavior. Based on these results, MUNASQA (tolerant) and TJ2049 (susceptible) were selected as the two more contrasting genotypes and incorporated into the EEAOC breeding program to develop a segregating population for genetic mapping of drought tolerance.

Meanwhile, in-depth phenotyping experiments were conducted to better understand the molecular and morphophysiological mechanisms involved in these genotypes' responses to drought. Moreover, we identify and statistically validate traits associated with drought-tolerance and yield-stabilization, aiming at their future use in drought-resilience breeding strategies such as genomic selection, especially in early developmental stages.

## Results and discussion

Here, we show differences in the molecular, morphophysiological, and biochemical responses of two contrasting soybean genotypes, MUNASQA (drought tolerant) and TJ2049 (drought susceptible), subjected to mild water deficit treatments in V3 (second open trefoil) and R5 (beans beginning to develop at one of the four uppermost nodes with a wholly unrolled leaf) phenological stages.

### Molecular insights

Transcriptional changes were assessed in MUNASQA and TJ2049 after 72 h of exposure to mild water deficit. From 38.658 transcripts analyzed, drought-stressed MUNASQA and TJ2049 plants exhibited 2952 and 1126 transcripts with significant changes (*P* < 0.05) in their expression levels (Suppl. Data 1), respectively. After an FDR = 0.1, 399 and 15 transcripts were assigned as MUNASQA and TJ2049 DEGs (Suppl. Data 2). The transcript loss detected in TJ2049 might be explained by the larger variation among replicates observed in the water deficit samples and measured as SD/mean ratio for each gene (Suppl. Table 1, Fig. S2). However, in a previous exploratory 454 sequencing experiment, TJ2049 showed significantly fewer DEGs than MUNASQA (data not shown).

**Figure 1:**
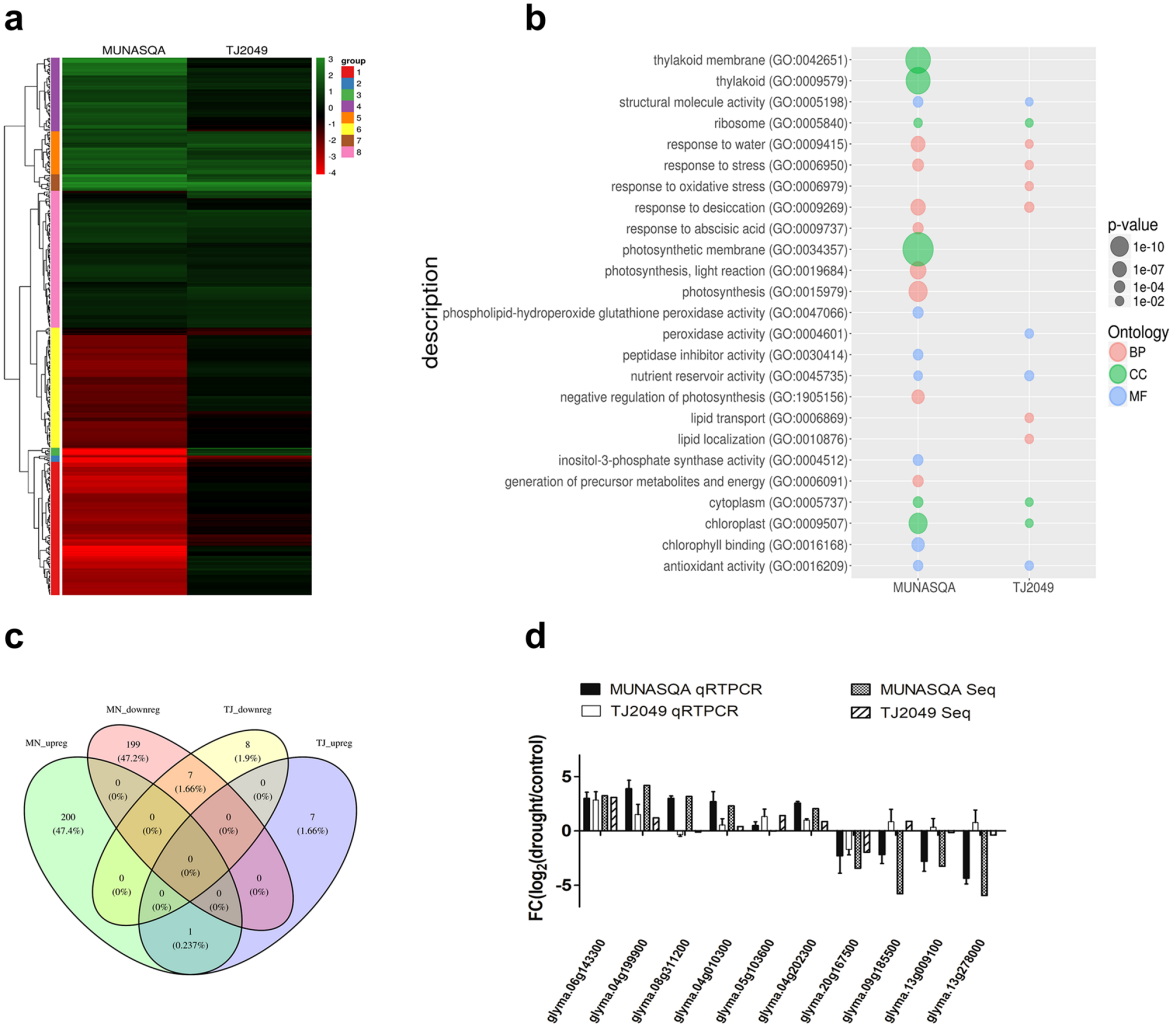
Transcriptomic analysis of MUNASQA and TJ2049 genotypes under drought. Heat-map of all DEGs for MUNASQA and TJ2049 in drought conditions. Scale color indicates green for up-regulation and red for downregulation (**a**). GO enrichment in MUNASQA and TJ2049 comprises biological processes (BP, in red), molecular function (MF, in blue), and cellular component (CC in green). Relevant categories showing enrichment of DEGs for both genotypes are depicted. GO terms were plotted after applying an FDR = 0.1 Bubble size correlates with enrichment factor values; for each bubble size, the P-value is indicated (**b**). Venn diagram for all DEGs in MUNASQA and TJ2049 under drought conditions. DEGs were plotted after applying an FDR = 0.1 (**c**). Validation by qRT-PCR of ten genes selected from RNA-Seq. Log_2_ fold change (log2FC) was calculated based on the comparison of drought *vs* control for each genotype (**d**). Three biological replicates were used, and the experiment was performed twice with similar results.

Large-scale transcriptional reprogramming has long been recognized as the first response to drought, initiating stress mitigation pathways and metabolic changes^14^. Moreover, the quickness to sense and respond to stresses could be essential for differentiating tolerant and susceptible genotypes. Here, after a mild drought, far from normal field levels, the genotypes exhibited dramatic changes on their transcriptional profiles and no observable phenotypic alteration. In the heat map (Fig. 1a), DEGs expression profiles were classified in 8 clusters (Suppl. Data 3), and almost 50% of MUNASQA DEGs showed repression under stress conditions, a difference reinforced by the Venn diagram (Fig. 1c).

Generally, in response to drought, plants initially trigger transcriptional control and hormone signaling, leading to metabolic adjustment for coping with low water availability. Cellular mechanisms such as water/ion uptake and transport, redox homeostasis, ROS scavenging, osmoregulation, and membrane protection are accompanied by physiological responses such as stomata regulation, root development, and protection of the photosynthesis machinery^15^. MUNASQA up-regulated several DEGs involved in these physiological responses (Suppl. Table 2). DEGs from "chlorophyll-binding" and "antioxidant activity" (MF category), "thylakoid", "thylakoid membranes" and "chloroplast" (CC category), and "photosynthesis", "response to water" and "response to desiccation" (BP category) (Fig. 1b), were identified. Overall, these results indicate a tightly regulated stress-response, growth, and water use mechanisms in drought-stressed MUNASQAs.

The expression of ten randomly selected DEGs by qRT-PCR (Fig. 1d) agreed with the MACE profile data for MUNASQA and TJ2049, showing a high similarity between both methodologies. Moreover, genes encoding detoxifying proteins like SOD, CAT, and APX were differentially regulated in both genotypes (Suppl. Table 3). Enzymes like these are stress-response indicators, frequently regulated at both transcriptional and post-transcriptional levels under drought^16^. We detected two up-regulated SOD genes under stress conditions in MUNASQA and, contrary to TJ2049, three down-regulated genes for APX and CAT. Accordingly, SOD activity measurements in R5 corroborated these differences in transcriptional activity between the two genotypes (Suppl. Table 4).

### Morphophysiological and biochemical phenotyping

MACE assay, by itself, does not fully explain the extent of MUNASQA and TJ2049 responses to the applied stress. Thus, extensive morphophysiological and biochemical measurements were performed to minimize the gap between the transcriptional regulation and phenotypical alterations observed. Here, 22 markers associated with stress-response, growth, and water-use were evaluated in both genotypes exposed to mild water deficit in V3 and R5 stages.

### Stress-response markers

An efficient antioxidant activity is crucial for withstanding low cellular water content, and its importance in plant drought tolerance has been extensively reported^17^. Here, substantial differences were found in MUNASQA and TJ2049 SOD, APX, POX, and CAT enzymes regulation over time, under water deficit, and independently of the phenological stage (Table 1). MUNASQA reached maximum activity for all enzymes after 4 d of water deficit, while the highest activity for TJ2049 occurred to 8 d after the stress onset, except for CAT. Noticeably, under non-stressed conditions, all enzymatic activities, excluding CAT, showed higher activity in the tolerant genotype than in the susceptible one, strengthening the hypothesis that TJ2049, compared to MUNASQA, presents delayed stress perception and response mechanisms. In fact, and according to Carvalho et al.^16^, a successful response to water deficit may depend not only on which enzymes are activated but also on the activation timing. Moreover, the general gene expression profile regarding these enzymes regulation was consistent with the biochemical results.

**Table 1:** Effect of mild water deficit on stress-response enzymatic markers measured in MUNASQA and TJ2049.

Genotype and treatment	SOD (Superoxide dismutase; µmol O_2_^−^ gDW^−1^ min^−1^)																APX (Ascorbate peroxidase; µmol Asa gDW^−1^ min^−1^)															
	ANOVA												Correlations				ANOVA												Correlations			
	V3 Stage						R5 Stage						V3 *vs* R5		*vs* Yield		V3 Stage						R5 Stage						V3 *vs* R5		*vs* Yield	
	0 DAS		4 DAS		8 DAS		0 DAS		4 DAS		8 DAS		r^2^		r^2^		0 DAS		4 DAS		8 DAS		0 DAS		4 DAS		8 DAS		r^2^		r^2^	
TJ2049 Control	60.98	**Aa**	58.02	**Aa**	61.25	**Ba**	75.64	**A**	78.89	**A**	65.07	**B**	0.62 (S)	***	− 0.12	ns	99.69	**Aa**	96.07	**Aa**	99.87	**Aa**	103.9	**Aa**	100.13	**Aa**	104.09	**Aa**	0.90 (S)	***	0.29 (W)	*
TJ2049 Stress	56.38	**Aa**	54.84	**Aa**	194.4	**Db**	73.82	**Aa**	91.78	**Bb**	153.1	**Dc**	0.93 (S)	***	0.17	ns	96.17	**Aa**	108.51	**ABa**	158.22	**Bb**	98.46	**Aa**	111.09	**Aa**	161.99	**Cb**	0.94 (S)	***	0.21	ns
MUNASQA Control	85.25	**Ba**	90.05	**Ba**	83.56	**Ca**	94.58	**Ba**	100.73	**Ba**	88.55	**C**	0.97 (S)	***	− 0.04	ns	120.9	**Ba**	126.42	**Ba**	117.69	**Aa**	133.01	**Ba**	139.08	**Ba**	129.48	**Ba**	0.97 (S)	***	0.25	ns
MUNASQA Stress	76.36	**Bb**	183.55	**Cc**	31.55	**Aa**	97.51	**Bb**	299.59	**Cc**	47.33	**Aa**	0.96 (S)	***	0.10	ns	121.84	**Ba**	205.1	**Cc**	174.46	**Bb**	134.87	**Ba**	227.02	**Cc**	193.11	**Db**	0.95 (S)	***	0.70 (S)	***
Standard Error	4.38		4.99		5.1		3.41		6.08		4.46						4.21		4.89		5.47		6.54		5.17		5.77					

	POX (Phenol peroxidase; µmol Purpurogalline gDW^−1^ min^−1^)																CAT (Catalase; µmol H_2_O_2_ gDW^−1^ min^−1^)															
	ANOVA												Correlations				ANOVA												Correlations			
	V3 Stage						R5 Stage						V3 *vs* R5		*vs* Yield		V3 Stage						R5 Stage						V3 *vs* R5		*vs* Yield	
	0 DAS		4 DAS		8 DAS		0 DAS		4 DAS		8 DAS		r^2^		r^2^		0 DAS		4 DAS		8 DAS		0 DAS		4 DAS		8 DAS		r^2^		r^2^	
TJ2049 Control	59.4	**Aa**	66.03	**Aa**	64.92	**Aa**	72.12	**Aa**	80.18	**Aa**	78.83	**Aa**	0.93 (S)	***	0.60 (S)	***	101.2	**Aa**	94.32	**Aa**	95.69	**Aa**	122.88	**Aa**	114.53	**Aa**	113.75	**Aa**	0.95 (S)	***	0.72 (S)	***
TJ2049 Stress	62.85	**Aa**	103.78	**BCb**	143.33	**Cc**	74.24	**Aa**	122.60	**BCb**	169.32	**Cc**	0.97 (S)	***	0.34 (W)	**	100.27	**Aa**	140.09	**Cb**	155.66	**Cc**	118.45	**Aa**	165.50	**Cb**	183.89	**Cc**	0.93(S)	***	0.46 (W)	***
MUNASQA Control	91.97	**Ba**	90.02	**ABa**	87.15	**ABa**	111.39	**Ba**	109.03	**ABa**	105.55	**ABa**	0.96 (S)	***	0.39 (W)	***	99.58	**Aa**	97.83	**Aa**	96.8	**Aa**	124.62	**Aa**	120.86	**Aa**	117.25	**Aa**	0.88(S)	***	0.49 (W)	***
MUNASQA Stress	98.87	**Ba**	121.89	**Cb**	91.09	**Ba**	119.39	**Ba**	147.18	**Cb**	109.99	**Ba**	0.94 (S)	***	0.27 (W)	*	97.42	**Aa**	120.93	**Bb**	127.54	**Bb**	117.63	**Aa**	146.02	**Bb**	154.01	**Bb**	0.90(S)	***	0.86 (S)	***
Standard Error	3.98		6.99		6.08		4.75		8.42		7.33						3.65		3.43		3.15		4.39		4.12		3.78					

	PRO (Proline; µg gFW^−1^)																MDA (Malondialdehyde; µmol gFW^−1^)															
	ANOVA												Correlations				ANOVA												Correlations			
	V3 Stage						R5 Stage						V3 *vs* R5		*vs* Yield		V3 Stage						R5 Stage						V3 *vs* R5		*vs* Yield	
	0 DAS		4 DAS		8 DAS		0 DAS		4 DAS		8 DAS		r^2^		r^2^		0 DAS		4 DAS		8 DAS		0 DAS		4 DAS		8 DAS		r^2^		r^2^	
TJ2049 Control	34.43	**Aa**	35.81	**Aa**	37.42	**Aa**	58.75	**Aa**	51.68	**Aa**	44.91	**Ab**	0.12	ns	0.73 (S)	***	6.09	**Ca**	6.89	**Ba**	6.05	**Ba**	9.75	**Ba**	9.42	**Ba**	9.67	**Ba**	0.90 (S)	***	0.71(S)	***
TJ2049 Stress	35.18	**Aa**	76.48	**Cb**	132.74	**Cc**	52.77	**Aa**	114.72	**Cb**	199.12	**Cc**	0.96 (S)	***	0.47 (W)	***	5.39	**Ba**	10.4	**Cb**	18.54	**Dc**	9.03	**Ba**	19.84	**Db**	29.67	**Dc**	0.91 (S)	***	0.48 (W)	***
MUNASQA Control	49.43	**Ba**	51.46	**Ba**	53.85	**Ba**	73.00	**Ba**	75.29	**Ba**	78.11	**Ba**	0.92 (S)	***	0.81 (S)	***	3.03	**Aa**	2.73	**Aab**	2.02	**Ab**	4.85	**Aa**	4.37	**Aa**	4.22	**Aa**	0.94 (S)	***	0.28 (W)	*
MUNASQA Stress	47.86	**Ba**	158.13	**Dc**	139.68	**Cb**	74.14	**Ba**	237.19	**Dc**	209.51	**Cb**	0.94 (S)	***	0.86 (S)	***	3.12	**Aa**	6.43	**Bb**	10.95	**Cc**	4.99	**Aa**	10.32	**Cb**	17.51	**Cc**	0.90 (S)	***	0.70 (S)	***
Standard Error	1.41		2.32		2.85		2.56		3.51		4.30						0.27		0.35		0.4		0.43		0.56		0.64					

	CHL (Chlorophylls; µg gFW^−1^)																CAR (Carotenoids; µg gFW^−1^)															
	ANOVA												Correlations				ANOVA												Correlations			
	V3 Stage						R5 Stage						V3 *vs* R5		*vs* Yield		V3 Stage						R5 Stage						V3 *vs* R5		*vs* Yield	
	0 DAS		4 DAS		8 DAS		0 DAS		4 DAS		8 DAS		r^2^		r^2^		0 DAS		4 DAS		8 DAS		0 DAS		4 DAS		8 DAS		r^2^		r^2^	
TJ2049 Control	368.74	**Aa**	497.61	**Bb**	591.12	**Cc**	442.48	**Aa**	578.53	**Bb**	699.35	**Cc**	0.96 (S)	***	0.37 (W)	**	138.00	**Aa**	151.76	**Ab**	141.39	**Aab**	179.40	**Aa**	190.29	**Ab**	183.81	**Aa**	0.86 (S)	***	0.95 (S)	***
TJ2049 Stress	376.60	**Ab**	418.14	**Ac**	216.02	**Aa**	451.92	**Ab**	489.77	**Ac**	259.22	**Aa**	0.92 (S)	***	0.13	ns	147.40	**Aab**	160.95	**Bb**	202.25	**Cc**	181.55	**Aa**	199.95	**Ab**	234.40	**Bc**	0.92 (S)	***	0.87 (S)	***
MUNASQA Control	371.00	**Aa**	490.32	**Bb**	589.85	**Cc**	445.20	**Aa**	594.18	**Bb**	707.82	**Cc**	0.85 (S)	***	0.76 (S)	***	139.65	**Aa**	153.81	**Ab**	180.31	**Bc**	256.62	**Bab**	248.24	**Ba**	262.93	**Cb**	0.89 (S)	***	0.92 (S)	***
MUNASQA Stress	374.50	**Ab**	377.12	**Ab**	311.25	**Ba**	449.40	**Ab**	452.54	**Ab**	373.5	**Ba**	0.88 (S)	***	0.13	ns	132.74	**Aa**	256.94	**Cb**	279.94	**Dc**	258.36	**Ba**	334.02	**Cb**	363.92	**Dc**	0.90 (S)	***	0.97 (S)	***
Standard Error	15.11		13.82		13.07		18.14		16.58		15.68						3.16		3.03		3.47		4.10		3.93		4.51					

SOD, APX, POX, and CAT activities, together with PRO, MDA, CHL, and CAR contents, were obtained from plants submitted to water deficit (Ψs = − 0.65 MPa) and well-watered treatments (Ψs = − 0.05 MPa) applied in V3 and R5 stages. Two independent experiments (n = 10 *per* genotype/treatment) were conducted, assessing parameters at 0, 4, and 8 d after stress (DAS) imposition. Additionally, 50 plants *per* genotype and the following treatments: 1: Control, 2: V3-Stress, and 3: R5-Stress, were harvested at physiological maturity to obtain relative yield. Average values followed by the same uppercase letter in the column and the same lowercase letter in the row do not differ statistically among them within each phenological stage, according to Tukey’s HSD test at 5%. The strength of association between markers evaluated in V3 and R5 stages (n = 240) and between markers and yield (n = 300) was measured by Pearson's correlation analysis adjusted by Bonferroni (*P* > 0.05 indicated as **ns**; *P* < 0.05 indicated as *; *P* < 0.01 ** and *P* < 0.001 ***). Correlation coefficients (*r*^2^) were classified as “**S**: Strong” (> ± 0.60) and “**W**: weak” (below ± 0.59).

Significant values are in bold.

Regarding PRO, one of the most common osmoprotectant in plants^18^, MUNASQA and TJ2049 accumulated the osmolyte in response to water deficit over time and in both V3 and R5 stages (Table 1). Several studies have demonstrated a direct correlation between high osmoprotectant accumulation and drought tolerance in many crops^19^. Here, the tolerant genotype MUNASQA exhibited a higher and more rapid accumulation of PRO after 4 d of water deficit at both phenological stages. In agreement with our results, a recent study in soybean reported higher PRO accumulation in the drought-tolerant genotype A5009 RG, compared with the drought-susceptible ADM50048^20^.

When analyzing MDA production, an indicator of lipid peroxidation and stress severity^21^, a higher and more rapid accumulation was detected in TJ2049 plants in response to water deficit, compared to MUNASQA (Table 1).

Drought also affects leaf pigments content^21^. Changes in photosynthetic pigments can alter various light-harvesting processes, while the accumulation of photoprotective compounds plays an essential role in preventing photo-oxidative damage^22^. Here, MUNASQA and TJ2049 showed alterations in pigment content under water deficit (Table 1). The CHL was significantly reduced over time due to stress in both genotypes, but this reduction was significantly lower in the tolerant one. Similar results were found in drought-tolerant maize that showed lower CHL reductions under stress than susceptible genotypes^23^. Regarding CAR levels, TJ2049 and MUNASQA showed an increased synthesis in response to drought, although the last one exhibited greater and faster accumulation.

According to our results, MUNASQA drought tolerance is strongly related to a rapid stress-sensing and response capacity and an efficient ROS (reactive oxygen species) scavenging system, both essential mechanisms for stress tolerance in numerous species^24^.

### Growth and yield markers

The ability to produce high seed yield or biomass under limited water access is considered the optimal indicator of drought tolerance in crops^25–27^. We evaluated the effect of mild water deficit in MUNASQA and TJ2049 growth and yield by monitoring changes in various markers related to biomass and seed production, including leaf area index (LAI), leaf area ratio (LAR), the net assimilation rate (NAR), relative growth rate (RGR), crop growth rate (CGR), relative yield and DSI (Table 2).

**Table 2:** Effect of mild water deficit on growth markers measured in MUNASQA and TJ2049.

Genotype and Treatment	LAI (Leaf Area Index)																LAR (Leaf Area Ratio; cm^−2^ g^−1^)															
	ANOVA												Correlations				ANOVA												Correlations			
	V3 Stage						R5 Stage						V3 *vs* R5		*vs* Yield		V3 Stage						R5 Stage						V3 *vs* R5		*vs* Yield	
	0 DAS		4 DAS		8 DAS		0 DAS		4 DAS		8 DAS		r^2^		r^2^		0 DAS		4 DAS		8 DAS		0 DAS		4 DAS		8 DAS		r^2^		r^2^	
TJ2049 Control	18.88	**Aa**	23.76	**Ab**	29.00	**Ac**	121.12	**Ba**	151.24	**Bb**	187.29	**BCc**	0.94 (S)	***	0.01	ns	54.30	**Cb**	42.41	**Ba**	50.49	**Bab**	11.17	**Aa**	14.40	**Ab**	16.45	**Ab**	0.44 (W)	***	− 0.03	ns
TJ2049 Stress	16.94	**Aa**	19.65	**Ab**	26.76	**Ac**	97.03	**Aa**	112.29	**Ab**	151.94	**Ac**	0.96 (S)	***	0.69 (S)	***	50.07	**Cb**	31.62	**Aa**	35.41	**Aa**	8.50	**Aa**	11.20	**Aa**	17.76	**Ab**	− 0.04	ns	− 0.66 (S)	***
MUNASQA Control	30.18	**Ba**	32.90	**Ba**	39.71	**Bb**	177.59	**Ca**	187.94	**Ca**	194.47	**Ca**	0.79 (S)	***	0.04	ns	83.73	**Bb**	58.07	**Ca**	49.40	**Ba**	18.60	**Ba**	18.38	**Ba**	16.58	**Aa**	0.36 (W)	***	− 0.04	ns
MUNASQA Stress	31.76	**Bab**	33.88	**Bb**	29.00	**Aa**	172.29	**Ca**	162.47	**BCa**	164.94	**ABa**	0.39 (W)	***	0.42 (W)	***	96.37	**Bb**	47.58	**Ba**	35.93	**Aa**	22.06	**Ba**	20.57	**Ba**	21.11	**Ba**	0.40 (W)	***	− 0.27 (W)	***
Standard Error	0.96		1.43		1.12		3.8		7.72		6.34						5.67		2.45		2.25		2.62		1.89		0.81					

	NAR (Net Assimilation Rate; g^−^^1^ cm^−2^ day^−^^1^)																RGR (Relative Growth Rate; g^−^^1^ g^−^^1^ day^−^^1^)															
	ANOVA												Correlations				ANOVA												Correlations			
	V3 Stage						R5 Stage						V3 *vs *R5		*vs *Yield		V3 Stage						R5 Stage						V3 *vs *R5		*vs *Yield	
	0 DAS		4 DAS		8 DAS		0 DAS		4 DAS		8 DAS		r^2^		r^2^		0 DAS		4 DAS		8 DAS		0 DAS		4 DAS		8 DAS		r^2^		r^2^	
TJ2049 Control	–		3.93	**Aa**	3.67	**Ba**	–		0.60	**Ba**	0.70	**Cb**	0.22	ns	− 0.03	ns	–		1.56	**Ca**	1.34	**Bb**	–		0.33	**Aa**	0.36	**Aa**	− 0.08	ns	− 0.11	ns
TJ2049 Stress	–		3.51	**ABa**	2.72	**Ab**	–		0.44	**Aa**	0.48	**Aa**	− 0.04	ns	0.01	ns	–		1.52	**Ca**	0.38	**Cb**	–		0.28	**Aa**	0.32	**Aa**	0.33	***	− 0.07	ns
MUNASQA Control	–		5.41	**Ca**	5.43	**Da**	–		0.64	**Ba**	0.72	**Cb**	0.10	ns	− 0.01	ns	–		2.69	**Ab**	3.24	**Aa**	–		0.30	**Aa**	0.34	**Aa**	− 0.45 (W)	ns	− 0.05	ns
MUNASQA Stress	–		4.51	**Ba**	5.04	**CDb**	–		0.63	**Bb**	0.54	**Ba**	0.17	ns	− 0.03	ns	–		1.91	**Bb**	1.36	**Ba**	–		0.32	**Aa**	0.33	**Aa**	0.22	ns	− 0.13	ns
Standard Error	–		0.13	–	0.12		–		0.04		0.09			–			–		0.13		0.10		–		0.09		0.05					

	CGR (Crop Growth Rate; g^−1^ cm^−2^ day^−1^)																															
	ANOVA												Correlations																			
	V3 Stage						R5 Stage						V3 *vs* R5		*vs* Yield																	
	0 DAS		4 DAS		8 DAS		0 DAS		4 DAS		8 DAS		r^2^		r^2^																	
TJ2049 Control	–		0.96	**Aa**	0.62	**Ab**	–		0.57	**Abb**	0.63	**Ca**	− 0.46 (W)	***	− 0.13	ns																
TJ2049 Stress	–		0.90	**Aa**	0.72	**Bb**	–		0.52	**Aa**	0.51	**Aa**	0.17	ns	0.02	ns																
MUNASQA Control	–		1.37	**Ba**	0.59	**Ab**	–		0.64	**Ca**	0.67	**Ca**	− 0.20	ns	− 0.05	ns																
MUNASQA Stress	–		1.27	**Ba**	0.67	**Abb**	–		0.60	**Bca**	0.58	**Ba**	0.33	ns	0.03	ns																
Standard Error	–		0.60	–	0.09		–		0.12		0.11																					

LAI, LAR, NAR, RGR and CGR were assessed in plants submitted to water deficit (Ψs = − 0.65 MPa) and well-watered treatments (Ψs = − 0.05 MPa) in V3 and R5 stages. Two independent experiments (n = 10 *per* genotype/treatment) were conducted, assessing parameters at 0, 4, and 8 d after stress (DAS) imposition. Additionally, 50 plants *per* genotype and the following treatments: 1: Control, 2: V3-Stress and 3: R5-Stress, were harvested at physiological maturity to obtain relative yield. Average values followed by the same uppercase letter in the column and the same lowercase letter in the row do not differ statistically among them within each phenological stage, according to Tukey’s HSD test at 5%. The strength of association between markers evaluated in V3 and R5 stages (n = 240) and between markers and yield (n = 300) was measured by Pearson's correlation analysis adjusted by Bonferroni (*P* > 0.05 indicated as **ns**; *P* < 0.05 indicated as *; *P* < 0.01 ** and *P* < 0.001 ***). Correlation coefficients (*r*^2^) were classified as “**S**: Strong” (> ± 0.60) and “**W**: weak” (below ± 0.59).

Significant values are in bold.

Total leaf surface area and LAI are strongly associated with canopy interception, evapotranspiration, and photosynthesis^28^. Here, independently of the phenological stage or water availability, MUNASQA plants exhibited a higher LAI compared to TJ2049, indicating a larger assimilatory capacity and, as a consequence, photosynthetic potential. In vegetative stages, a higher LAI denotes a more rapid canopy development, favoring greater and faster soil coverage and thus less water loss from direct evaporation. In general, drought suppresses leaf initiation and growth and consequently affects LAI^29^. Therefore, a decrease in LAI is expected in plants exposed to water scarcity. As expected, MUNASQA and TJ2049 plants showed a reduction in LAI in response to drought, more noticeable after 8 d of stress and in the R5 stage.

Drought also alters the LAR: the leaf area development in relation to the total biomass produced^30^. Here, water deficit affected the LAR in both genotypes, but only during the vegetative stage. The highest ratio between plant leaf area and biomass is reached at the beginning of the plant life cycle^31^, which explains the highest LAR values at the first sampling day during the vegetative stage.

In MUNASQA and TJ2049, the relationship between leaf area expansion and the biomass produced over time (NAR) was also reduced due to water deficit at both phenological stages. Changes in photosynthetic efficiency were more significant at V3, where both genotypes exhibited greater NAR values. Noticeably, well-watered MUNASQA plants showed a significantly higher NAR than TJ2049 ones, while, in response to stress, MUNASQA’s NAR increased in contrast to TJ2049 values, which were reduced. Although not as accentuated, a similar pattern was observed in plants exposed to water deficit in R5. Here, LAI, LAR, and NAR results indicated that, in response to drought, MUNASQA plants regulated photosynthates allocation to leaves and the maintenance of photosynthetic efficiency more efficiently than TJ2049 ones.

In addition, the relative growth rate (RGR) or biomass produced over time was significantly reduced by water deficit in the vegetative stage (Table 2). Interestingly, the reduction in RGR was more pronounced in TJ2049 plants. Regarding CGR, significant differences were observed in the V3 stage after 8 d of drought, while in R5 were detected after 4 d.

**Figure 2:**
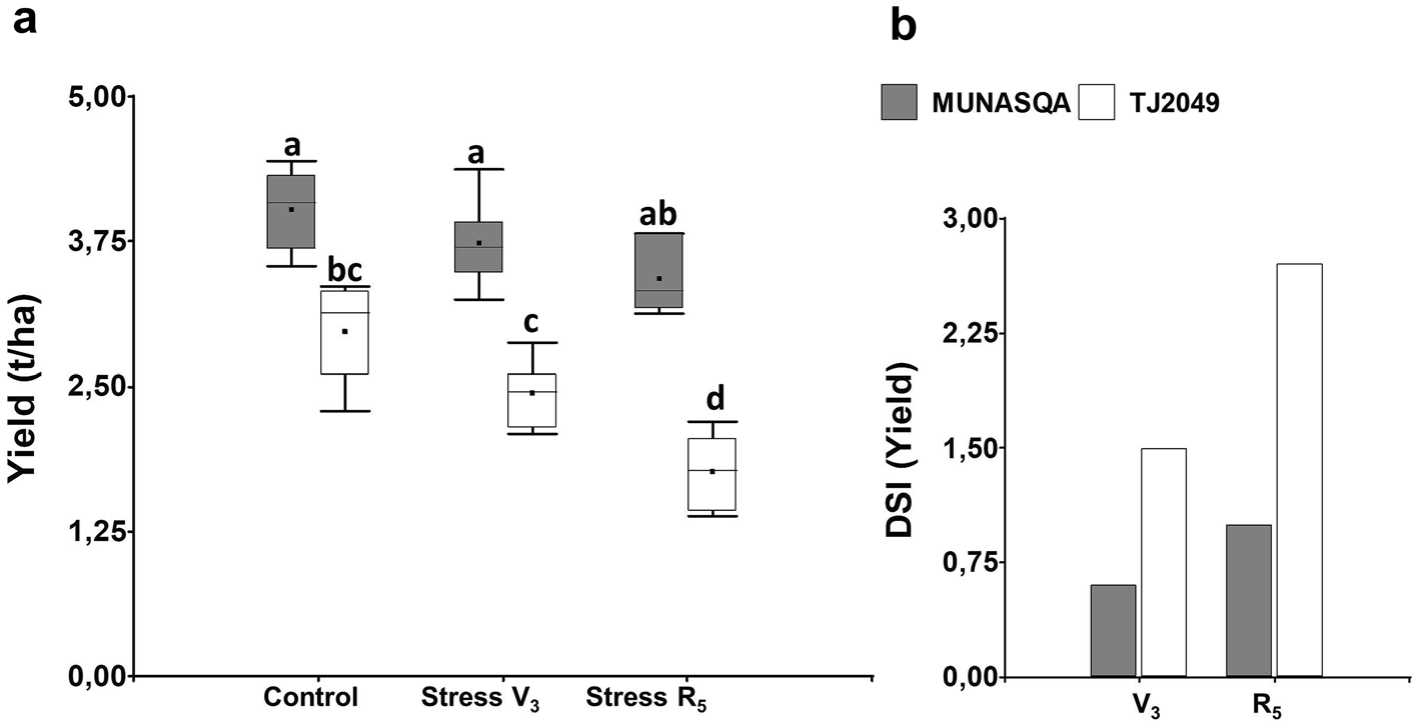
Effects of mild water deficit in MUNASQA and TJ2049 yield and yield-DSI. Yield in well-irrigated (Ψs = − 0.05 MPa) and drought-stressed (Ψs = − 0.65 MPa) V3 and R5 (**a**). Yield-DSI for each genotype phenotyped in V3 and R5 (**b**). Different letters indicate significant differences at *P* < 0.05 (two-way ANOVA). Error bars represent SE from independent experiments, n = 300 *per* trial.

These results agree with the differences in yield and yield-DSI exhibited by MUNASQA and TJ2049 after water-deficit treatments in V3 and R5 (Fig. 2). According to these findings, when drought was applied in the V3 stage, TJ2049 showed a distinct but not significant yield penalty and a yield-DSI considerably higher than MUNASQA. Moreover, after a mild water deficit in R5, a highly moisture-sensitive phenological stage, TJ2049 exhibited the largest yield loss and a significantly higher yield-DSI than MUNASQA. Results that also agree with the ones reported by Pardo et al.^13^.

### Water use markers

Maintaining tissue/cellular water content and/or metabolic activity at low water potentials are physiological strategies to survive drought^32^. Traits like pubescence, leaf thickness, stomatal density, closure, slow wilting, canopy temperature, RWC, and WUE are essential to determining drought tolerance in plants. Thus, all these water-use parameters were assessed in MUNASQA and TJ2049 in response to drought.

After 21 d of water deficit, the genotypes exhibited drought-induced adaptations in leaf morphology traits^33^, such as stomata, trichrome density, and leaf thickness (Table 3). As expected, the stomatal density was substantially altered in response to drought. Here, TJ2049 plants exhibited a considerable decrease, while MUNASQA ones showed an 89 and 65% increase on abaxial and adaxial leaf surfaces. Moreover, no changes in pubescence were observed on TJ2049 plants, while in MUNASQA, the trichrome density was increased, especially in the abaxial leaf surface. In response to drought, plants can reduce their stomatal size, density, or aperture and develop higher pubescence^34^, mainly on the abaxial surface^35^. The increase in MUNASQA’s stomatal density could indicate the genotype’s ability to produce smaller but denser stomata and, therefore, reduce transpiration by a quicker onset of stomatal regulation. This modification, combined with denser trichomes, could enhance the boundary layer resistance, increase the air’s moisture outside the stomata and minimize water loss during drought, precluding significant growth penalties in terms of photosynthetic activity.

**Table 3:** Effect of mild water deficit on leaf morphology of MUNASQA and TJ2049.

Genotype and	Stomatal density				Trichome density				Leaf thickness (µm)		Stomatal aperture (µm)	
Treatment	Abaxial surface (mm^2^)		Adaxial surface (mm^2^)		Abaxial surface (mm^2^)		Adaxial surface (mm^2^)					
TJ2049 Control	219.14	**C**	173.05	**C**	0.82	**A**	0.49	**A**	158.06	**B**	3.48	**D**
TJ2049 Stress	200.05	**B**	87.21	**B**	1.07	**A**	0.30	**A**	165.29	**C**	0.97	**B**
MUNASQA Control	186.32	**A**	52.05	**A**	2.29	**B**	0.81	**B**	158.12	**B**	2.84	**C**
MUNASQA Stress	351.66	**D**	79.56	**B**	3.22	**C**	1.51	**C**	149.59	**A**	0.45	**A**
Standard Error	2.62		2.38		0.11		0.07		1.71		0.07	

LT, TD_AB, TD_AD, ST_AB, SD_AD, and stomatal aperture were assessed in plants submitted to water deficit (Ψs = − 0.65 MPa) and well-watered treatments (Ψs = − 0.05 MPa) in R5 stage (except for stomatal aperture applied in V3). For LT, SD_AB, SD_AD, TD_AB and TD_AD, an independent experiment (n = 5 *per* genotype/treatment) was conducted, assessing parameters at 3, 10 and 21 days after stress (DAS) imposition. Here we showed the data corresponding to 21 DAS (n = 10 measured *per* sample). For stomatal aperture, three independent experiments (n = 40 stomatal measurements *per* genotype/treatment) were conducted, and the stomata evaluation was performed 72 hs after stress imposition. Average values followed by the same uppercase letter do not differ statistically according to Tukey’s HSD test at 5%.

Regarding leaf thickness, no significant differences were detected under well-irrigated conditions. However, after 21 d of water deficit, MUNASQA leaves were considerably thinner, while TJ2049 ones were thicker. The knowledge about the links between leaf morpho-anatomy and its function under non-stressed/stressed conditions is relatively poor, especially for traits like leaf thickness^36^ that is strongly related to transpiration^37^ and reported by some authors as a drought-tolerance trait that maintains turgor pressure and enhances photosynthesis^38^. Yet, this feature, only apparent in TJ2049 plants under water deficit, could not be associated with transpiration adjustments, photosynthesis increase, or any other drought-tolerant feature.

**Figure 3:**
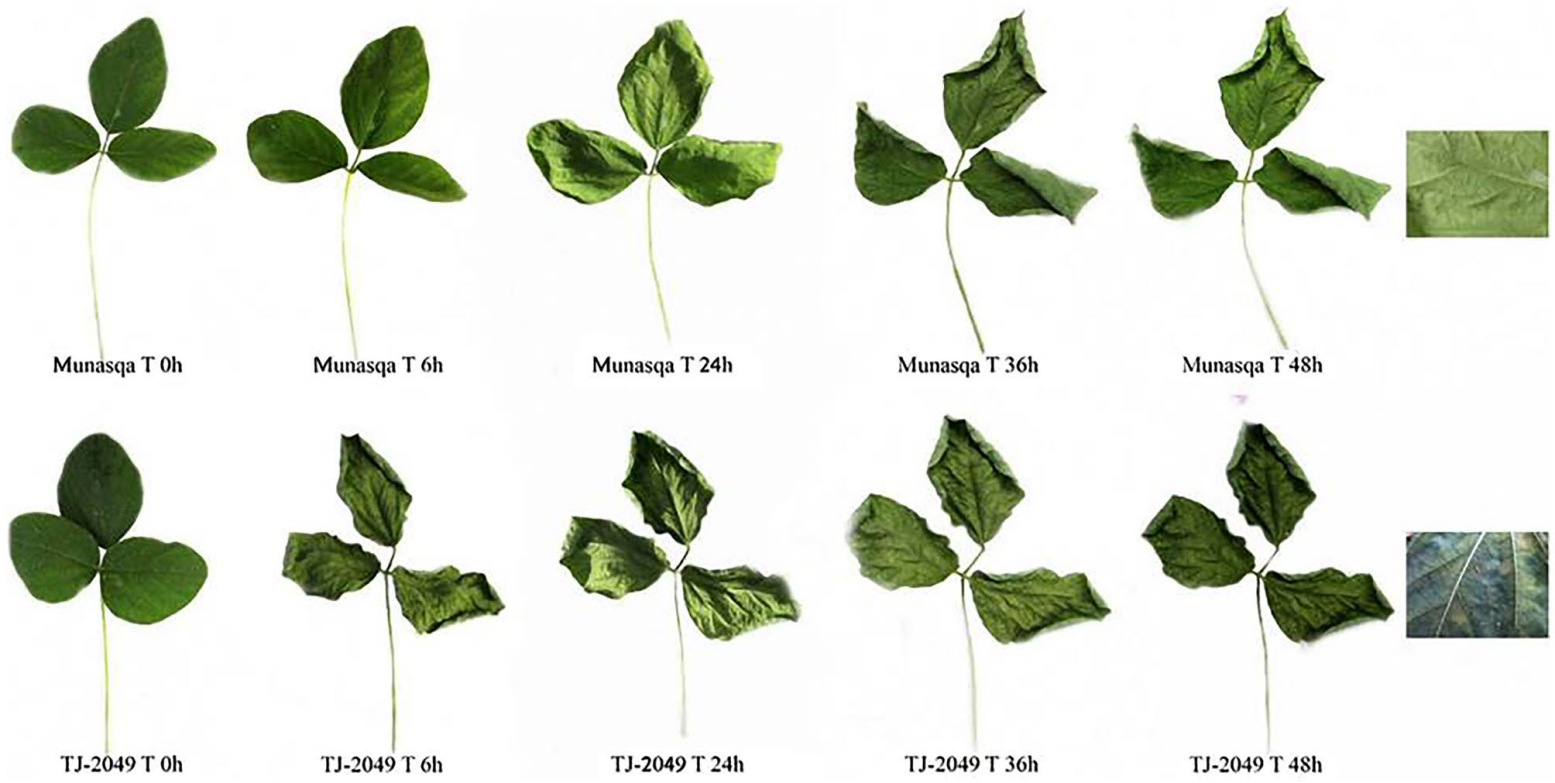
MUNASQA and TJ2049 response to wilting air desiccation. Whole leaves (n = 6), collected from R5 plants, were exposed to air desiccation at 32 °C and photographed after 0, 6, 24, 36, and 48 h to evaluate the appearance of wilting symptoms.

Meanwhile, the regulation of stomatal aperture reinforces the drought-tolerant character of MUNASQA. Under non-stressed conditions, the susceptible TJ2049 showed more opened stomata (⁓22% more than MUNASQA). Moreover, after 3 d of water deficit in V3, this difference was increased to almost ⁓50% of stomatal aperture (Table 3). Although stomata represent a small percentage of the leaf lamina, large amounts of water evaporate through them^33^. Thus, the lack of stomatal control in TJ2049 might explain the fast-wilting phenotype in response to air desiccation (Fig. 3). Measuring the water loss of detached leaves is a selection method for drought tolerance^39^. Here, R5 leaves of both genotypes were removed and air-dried. After 48 h, MUNASQA exhibited a greener, healthier and slow-wilting phenotype, previously linked to drought tolerance in studies with soybean cultivars^17^.

MUNASQA water-saving behavior was also confirmed through parameters such as RWC and WUE, strongly regulated under drought (Table 4). The RWC, a time-specific measurement of the hydric status of a plant, is considered a physiological character recommended for drought-tolerance selection^40^. In V3 and R5 stages, well-irrigated MUNASQA plants showed higher RWC than TJ2049, even with a smaller canopy. As expected, under water deficit, the RWC values were reduced by 14% in MUNASQA and 20% in TJ2049, indirectly confirming the effectiveness of the stress imposed.

**Table 4:** Effect of mild water deficit on water-use physiological markers measured in MUNASQA and TJ2049.

Genotype and Treatment	RWC (Relative Water Content; %)																WUE (Water Use Eficiency; g kg^−1^)															
	ANOVA												Correlations				ANOVA												Correlations			
	V3 Stage						R5 Stage						V3 *vs* R5		*vs* Yield		V3 Stage						R5 Stage						V3 *vs* R5		*vs* Yield	
	0 DAS		4 DAS		8 DAS		0 DAS		4 DAS		8 DAS		r^2^		r^2^		0 DAS		4 DAS		8 DAS		0 DAS		4 DAS		8 DAS		r^2^		r^2^	
TJ2049 Control	74.54	**Aa**	78.24	**Ca**	80.33	**Ca**	71.89	**Aa**	81.46	**Cb**	78.94	**Cab**	0.87 (S)	***	–	–	6.54	**Ca**	5.66	**Bb**	7.02	**Bc**	7.89	**Cb**	6.60	**Bc**	8.09	**Bb**	0.89 (S)	***	− 0.44 (W)	**
TJ2049 Stress	72.87	**Ab**	58.41	**Aa**	59.01	**Aa**	75.22	**Ab**	53.82	**Aa**	56.40	**Aa**	0.96 (S)	***	–	–	5.87	**Bb**	4.59	**Aa**	4.76	**Aa**	7.47	**Cb**	5.43	**Aa**	5.48	**Aa**	0.95 (S)	***	0.82 (S)	***
MUNASQA Control	83.42	**Ba**	80.07	**Ca**	85.37	**Ca**	82.89	**Ba**	85.40	**Ca**	88.65	**Ca**	0.90 (S)	***	–	–	5.16	**Aa**	5.72	**Bb**	6.52	**Bc**	6.23	**Aa**	6.77	**Bb**	7.51	**Bc**	0.91 (S)	***	− 0.78 (S)	***
MUNASQA Stress	83.08	**Bb**	68.16	**Ba**	65.76	**Ba**	80.55	**Bb**	64.36	**Ba**	60.30	**Ba**	0.94 (S)	***	–	–	5.42	**Aa**	7.68	**Cb**	8.58	**Cc**	7.03	**Ba**	8.24	**Cb**	7.91	**Bc**	0.96 (S)	***	0.84 (S)	***
Standard Error	1.90		1.85		1.88		2.20		2.04		3.07						0.48		0.24		0.54		0.38		0.18		0.49					

	CTD (Canopy temperature depression; ^o^C)																															
	ANOVA												Correlations																			
	V3 Stage						R5 Stage						V3 *vs* R5		*vs* Yield																	
	0 DAS		4 DAS		8 DAS		0 DAS		4 DAS		8 DAS		r^2^		r^2^																	
TJ2049 Control	3.11	**Ba**	2.97	**Ca**	3.21	**Ca**	3.37	**Ba**	3.50	**Ca**	3.25	**Da**	0.93 (S)	***	0.70 (S)	***																
TJ2049 Stress	2.86	**Bb**	1.58	**Ba**	1.87	**Ba**	3.52	**Bc**	1.54	**Bb**	1.02	**Ca**	0.91 (S)	***	− 0.79 (S)	***																
MUNASQA Control	1.27	**Aa**	1.19	**Ba**	1.33	**Ba**	1.77	**Aa**	1.46	**Ba**	1.61	**Ba**	0.95 (S)	***	0.76 (S)	***																
MUNASQA Stress	1.43	**Ab**	− 1.32	**Aa**	− 1.39	**Aa**	1.60	**Ab**	− 0.94	**Aa**	− 1.12	**Aa**	0.97 (S)	***	− 0.85 (S)	***																
Standard Error	0.28		0.30		0.37		0.17		0.22		0.40																					

RWC, WUE and CTD were assessed in plants submitted to water deficit (Ψs = − 0.65 MPa) and well-watered treatments (Ψs = − 0.05 MPa) in V3 and R5 stages. Two independent experiments (n = 10 *per* genotype/treatment) were conducted, assessing parameters at 0, 4, and 8 d after stress (DAS) imposition. Additionally, 50 plants *per* genotype and the following treatments: 1: Control, 2: V3-Stress and 3: R5-Stress, were harvested at physiological maturity to obtain relative yield. Average values followed by the same uppercase letter in the column and the same lowercase letter in the row do not differ statistically among them within each phenological stage, according to Tukey’s HSD test at 5%. The strength of association between markers evaluated in V3 and R5 stages (n = 240) and between markers and yield (n = 300) was measured by Pearson's correlation analysis adjusted by Bonferroni (*P* > 0.05 indicated as **ns**; *P* < 0.05 indicated as *; *P* < 0.01 ** and *P* < 0.001 ***). Correlation coefficients (*r*^2^) were classified as “**S**: Strong” (> ± 0.60) and “**W**: weak” (below ± 0.59).

Significant values are in bold.

Water use efficiency (WUE), referring to the biomass produced *per* water unit, has been widely used as a breeding target in many rainfed crops, including soybean. Conservative water-use strategies are associated with high leaf WUE^34^. In agreement, and contrarily to TJ2049, V3 and R5 MUNASQA plants showed a gradual increase of WUE in response to water deficit that agrees with the tighter regulation of stomatal movements and the reduced water loss observed in the genotype (Table 4). Moreover, considering the discrete NAR reduction and the maintenance of a ~ 70% RWC under water deficit, we hypothesize that MUNASQA may display a stomatal control based on partial or total/partial closure intervals, therefore reducing transpiration and saving water through a smaller gas exchange (potential photosynthesis) penalty.

The CTD, regarding plant canopy temperature difference with the surrounding air, is considered a surrogate trait for stomatal conductance and a good indication of plant transpiration rate^41^. As expected, in response to drought, MUNASQA plants evidenced lower CTD values, a finding that supports the stomatal aperture results and strongly suggests the genotype water-saving behavior. Plants with higher stomatal conductance transpire more and thus maintain a cooler canopy^42^. Thus, in TJ2049 stressed-plants, the high and positive CTD confirmed a higher stomatal aperture and transpiration rate that agrees with a water-spender behavior. Moreover, TJ2049 also presented higher transpiration rates in unstressed conditions. Finding that could be evidence of a natural and predisposing difference between tolerant and susceptible genotypes.

### Markers selection

Identifying and exploiting phenotyping markers will improve selection strategies for drought tolerance in legumes crops^17^. However, to successfully implement markers in a breeding program, it is imperative to validate their (i) accuracy, (ii) feasibility, and (iii) strength of association with the desired trait. To further understand the marker’s contribution to drought tolerance and yield stabilization in MUNASQA and TJ2049, Principal Component Analysis (PCA) was performed.

**Figure 4:**
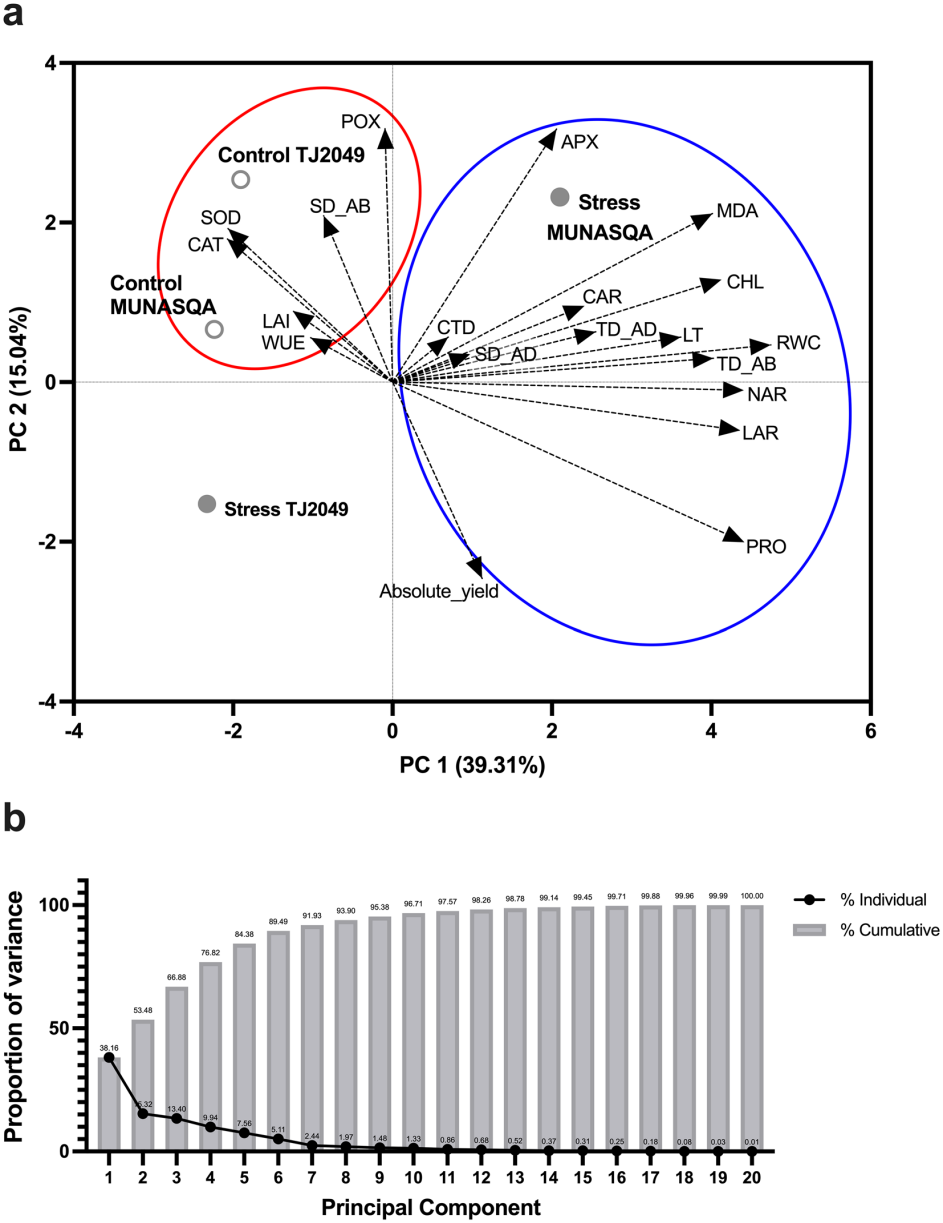
PCA for all the morphophysiological markers evaluated in MUNASQA and TJ2049 genotypes.

A first PCA was conducted for all the morphophysiological parameters evaluated together with absolute yield (Fig. 4). Here, data corresponding to MUNASQA stress was separated from the rest of the treatments and genotype in Principal Component (PC) I, showing the biggest dissimilarity (Fig. 4a). Meanwhile, TJ2049 stress data were separated in PC II. However, when comparing the proportion of variance explained by the different PC (Fig. 4b), the first two only explained 53,48%. Moreover, no clear association was observed between the absolute yield and the rest of the parameters, although PRO and LAR exhibited some positive relation.

In PCAs, an increase in the number of comparable variables will reduce the proportion of variance explained by those variables. Therefore, to discriminate which markers better explain the variability between genotypes and treatments, independent PCAs were conducted using subsets of parameters grouped by biological processes in (i) “stress response”, (ii) “growth” and (iii) “water use” categories (Fig. S3). In addition, markers evaluated at V3 and R5 stages were measured by Pearson's correlation to determine their strength of association between the phenological stages (accuracy) and yield stabilization (desired trait) (Tables 1, 2, and 4).

Biochemical parameters such as enzymatic and non-enzymatic ROS scavengers, leaf pigments, and MDA have been confirmed as adaptive responses to desiccation stress, frequently used for selecting plant genotypes under drought^43^. The PCA results showed clear discrimination of MUNASQA and TJ2049 drought responses (Fig. S3a). Here, the first two principal components (PC) explained 96.6% of total variation (PC1 = 75.0% and PC2 = 21.6%). Data were clustered by irrigation treatment in PC1, suggesting that these markers are indicators of phenotypic plasticity. The CHL was associated with well-irrigated plants, while all enzymes, MDA, CAR, and PRO, were related to drought stress.

All these markers showed high accuracy between phenological stages due to significant (*P* < 0.001), strong (*r*^2^ over 0.88), and positive correlations (Table 1). However, the correlation with yield showed inconsistent outcomes. PRO, MDA, and CAR were significant and positively correlated with yield. Meanwhile, except SOD, all enzymes exhibited significant and positive correlations that were too variable in the strength of association and could not be linked with a specific genotype or treatment. Thus, we considered these “stress-response” markers suitable for discriminating susceptible/resistant responses during early drought-tolerance screenings in soybean. Still, their use as breeding traits is limited due to the high environmental effect. Interestingly, a report^20^ found that PRO and CHL were suitable markers for ranking soybean genotypes in response to drought in vegetative stages (5 days after emergence). At the same time, MDA could be useful during R5 as a sensitivity trait.

In legumes, features like LAI, smaller leaf area, leaf area maintenance, and dry matter partitioning have been used to screen for drought tolerance^17^. Here, in the “growth” PCA (Fig. S3b), the first two PC explained 93.0% of total variation (PC1 = 73.0% and PC2 = 20.0%). In PC1, data were clustered by genotype, suggesting that these markers explained differences between MUNASQA and TJ2049 growth responses on their intrinsic genetic variability. Moreover, the markers analyzed were only associated with MUNASQA, LAR, and RGR associated with stressed plants. However, only LAI and LAR showed a weak correlation between phenological stage and yield (Table 2).

Water-saving features like denser leaf pubescence, a higher number of stomata, warmer canopies, RWC maintenance, and increased WUE have been associated with drought tolerance in legumes and applied in drought-resistance breeding^17^. Here, “water-use” markers contributed the most to discriminating drought-tolerant and susceptible responses. In the two PCA performed, one for physiological markers and the other for morphological ones, data were clustered by genotype in PC1; thus, these markers are indicators of genetic variability. In CTD, RWC and WUE PCA (Fig. S3c), the first two PC explained of total variation 87.7% (PC1 = 54.6% and PC2 = 33.1%). Markers RWC and WUE (associated with MUNASQA) and CTD (associated with TJ2049) showed significant (*P* < 0.001), strong (*r*^2^ over 0.87), and positive correlations between phenological stages (Table 4). Moreover, WUE and CTD were significantly associated with yield, showing positive and negative correlations depending on the water treatments applied (Table 4). In the PCA made with morphological “water-use” markers, the first two PC explained 96.6% of total variation (PC1 = 73.8% and PC2 = 22.8%) (Fig. S3d). Here, pubescence and stomata abaxial density was strongly related to MUNASQA. Although the data were insufficient to execute good correlation analysis, these morphological markers have been demonstrated as clear indicators of water-saving strategies in legumes^34^.

A good drought-tolerance marker linked to yield stabilization must also be accurate, cost-effective, if possible, non-destructive, and easily measurable. Hence, after evaluating marker accuracy (amid phenological stages) and assessing which ones better explained the phenotypic variability between genotypes and treatments, a final selection was performed by cost-feasibility (CF) and statistical weight (SW) rankings. Based on the CF values, the degree of complexity and cost to assess each indicator, nine markers were further selected, encompassing the categories 1 (easy and cheap) and 2 (easy and expensive) (Table 5). Subsequently, after SW re-selection, based on the percentage of variability among genotypes and treatments explained by each parameter, four markers remained with “High” SW in both PC.

**Table 5:** Selection of phenotyping markers according to their CF and SW.

Cost-feasibility (CF)	Marker selected by CF	Statistical weight (SW)		Marker reselected by SW
		PC 1	PC 2	
1	LAI	High	Low	-
2	SD_AB	**High**	**High**	**Sel**
2	SD_AD	**High**	**High**	**Sel**
2	TD_AB	**High**	**High**	**Sel**
2	TD_AD	High	Low	-
1	WUE	High	Low	-
2	CTD	**High**	**High**	**Sel**
2	MDA	Low	High	-
2	CAR	High	Low	-

Markers with **CF** of **1** or **2** and High SW in both autovectors were selected.

Significant values are in bold.

Determine which markers are more suitable to assess drought tolerance often represents a challenge and depends on the researcher's criteria, e.g., whether tolerance is based on yield maintenance or intrinsic mechanisms that ensure plant survival at the expense of productivity. During this research, we assessed numerous parameters associated with plant performance and stress responses, aiming to identify a small group of markers related to yield stabilization, stress tolerance, or both, and if possible, non-destructive and easily measurable. Often, some markers are highly accurate (stable during the plant life cycle) but expensive, laborious, and/or time-consuming (low throughput). Thus, without disregarding the importance of accuracy, we also consider markers that are cost-efficient and can be assessed in a high throughput manner as strong candidates for drought-tolerance phenotyping.

During this research, the selected markers were (i) stomatal density on the adaxial and (ii) abaxial leaf surface, (iii) trichrome density on the abaxial side, and (iv) CTD. These four traits were chosen as the most efficient phenotyping markers for drought tolerance due to their high accuracy, strong association to water-saving strategies under drought, high cost-efficiency (affordable and easily measured), and non-destructive assessment, therefore ideal for high throughput screening using high-resolution imaging.

Furthermore, beyond the contributions to our soybean breeding program, we must highlight the practical applications of the in-depth phenotyping results. Overall, these findings (i) confirmed the effectiveness of the methodologies used during the research (e.g., drought imposition, sampling times), (ii) corroborate its successful application in early phenological stages, and (iii) strength the usefulness of MUNASQA and TJ2049 as model genotypes for genetic mapping studies. In this context, our group developed a segregating population of 280 F6 RIL (recombinant inbred lines) families that will be mapped for drought tolerance with SSR (simple sequence repeats or microsatellites) and SNPs (single nucleotide polymorphism) selected based on the four markers chosen in this research (stomatal and trichome densities and CTD).

## Methods

### Experimental approach

The response of MUNASQA and TJ2049 to mild water deficit applied in the R5 stage (beans beginning to develop at one of the four uppermost nodes with a wholly unrolled leaf) was assessed through transcriptional and leaf morphology analysis. Subsequently, comparative studies were performed to determine the genotype's response to water deficit imposed in V3 and R5 stages. Next, all markers assessed were analyzed according to their strength of association between phenological stages and yield, then were ranked by statistical weight and cost-feasibility (Fig. S1).

### Plant material and growth conditions

All experiments were conducted in greenhouse conditions at the Estación Experimental Agroindustrial Obispo Colombres (EEAOC), Las Talitas, Tucumán, Argentina (S26° 50′, W65° 12′). Seeds of MUNASQA and TJ2049 were inoculated with *Bradyrhizobium japonicum* E109 strain (9 × 109 viable cells kg^−1^ of seeds) and sown in 4 L plastic pots (diameter: 18 cm, height: 21 cm) filled with GrowMix® Multipro commercial substrate (Terrafertil S.A., Argentina). Topsoil was covered with perlite to minimize water evaporation. Pots were weekly rearranged to minimize environmental effects. At the V1 stage, open leaf at the unifoliate node^44^, two homogeneous plants *per* pot were left. Comparative trials were performed at two phenological stages according to Fehr et al.^44^: V3 (second open trefoil) and R5 (beans beginning to develop at one of the four uppermost nodes with a wholly unrolled leaf). During all the experiments, environmental variables were assessed with sensors every 15 min, then recorded and averaged in data loggers (Cavadevices.com, Buenos Aires, Argentina) (Suppl. Table 5).

### Irrigation treatments

The volumetric water content (VWC) of each pot was estimated according to Pereyra-Irujo et al.^45^, and the relationship between VWC and water potential (Ψs) was determined^46^. Pots were maintained at 22% of VWC (Ψs = − 0.05 MPa) through daily watering until stress onset. According to Pardo et al.^13^, the water deficit was applied by maintaining the pots at 14% of VWC (Ψs = − 0.65 MPa) for ten days. The desired Ψs was reached in 2–3 days. At the end of stress, plants were fully watered until harvest. The Ψs was daily monitored and recorded. Corrections for soil water status were made by weighing two plants *per* genotype and treatment every 3 days. The plant water status was monitored through the RWC^47^ to ensure stress occurrence.

All drought experiments were carried out for three consecutive years, always applying the previously described irrigation treatments. Water deficit in V3 and R5 phenological stages was imposed in independent plant sets; the sections below detailed the sampling process.

### Experiments

#### MACE-transcriptomic analysis and validation

Three biological replicates *per* treatment (Control and Stress) and genotype (MUNASQA and TJ2049) were collected from R5 plants after 72 h of water deficit (n = 12), and RNA from fully mature expanded leaf between nodes 5 to 7 node was isolated for transcriptional analysis.

MACE-Seq libraries and sequencing were performed on an Illumina NextSeq500 machine (1 × 75 bp reads). The conversion was made with bcl2fastq2 software (version 2.19.1), and the cleaning of duplicate sequences was performed with "TrueQuant". In MACE-seq, the TrueQuant barcodes each DNA molecule before PCR amplification. As each barcode-template combination is statistically unique, PCR-duplicates can be identified and eliminated from the dataset to prevent PCR bias. Bases with low sequencing quality were clipped. Next, reads were mapped into genome version “Gmax_275_v2.0.fa” of soybean downloaded with standard parameters from Phytozome.net and Bowtie2 (version 2.2.4). Then, expression analysis was performed by in-house scripts and DESeq2 (R-package).

DEGs were defined at an FDR of 0.1 and listed as either up or down-regulated. A heat map plot was generated using R software (version 3.4.1). Then, hierarchical clustering was applied by considering a cut-off threshold of 8 expression profiles (clusters). Venn diagrams were depicted using the VennDiagram R package.

A GO enrichment analysis was performed using the topGO R package^48^, while the GO annotation file was extracted from agriGO website^49^. Each GO term, containing at least two DEGs, was analyzed by Fisher's exact test. The resulting P values were corrected by FDR multiple testing approach. GO terms with an FDR lower than 0.1 were considered for further analysis.

Ten DEGs were randomly selected and measured by qRT-PCR assays (Applied Biosystems) to validate MACE results. F-BOX gene was used as an internal reference to standardize the expression of target genes, and the ratio between treatments was calculated according to^50^. All primers used are listed in Suppl. Table 6. Data analysis and primer efficiencies were obtained using LinReg PCR software^51^. Relative expression ratios and statistical analysis were performed using fgStatistics software interface^52^. The cut-off for statistically significant differences was set as *P* < 0.05, indicated as *.

#### Antioxidant measurements

Additionally, antioxidant proteins encoded by DEGs detected in MACE were analyzed. Five biological samples were collected *per* treatment and genotype (n = 20). The enzymatic extraction was performed according to Singh et al.^53^. The activities of superoxide dismutase (SOD, EC 1.15.1.1)^54^, ascorbate peroxidase (APX, EC 1.11.1.11)^55^, phenol peroxidase (POX, EC 1.11.1.7)^56^ and catalase (CAT, EC 1.11.1.6)^57^ were measured, as well as the total soluble protein content^58^.

#### Leaf morphology measurements

Changes in leaf thickness (LT), adaxial and abaxial stomatal and trichrome densities (SD_AD, SD_AB, TD_AD and TD_AB) were assessed in leaves between nodes 4 to 7 of MUNASQA and TJ2049 R5 plants after 3, 10 and 21 d of water deficit. Five samples *per* genotype and treatment (n = 20) were taken and fixed in FAA (10% formalin, 5% acetic acid, 50% ethyl alcohol). Diaphanised sections of the central leaflet were used for superficial observations. Different standard colorations were applied according to D’Ambrogio de Argüeso (1986)^59^. Staining samples were visualized in a Leica DM500 optical microscope and photographed with an Arcano (5 Mpx) camera (10 measurements *per* sample, n = 200).

#### Stomatal aperture measurements

According to Gudesblat et al.^60^, stomatal apertures were measured in three independent assays, using MUNASQA and TJ2049 plants submitted to 72 h of water deficit in the V3 stage. The aperture of 40 stomata *per* treatment and genotype (n = 120) was measured in each experiment.

#### Wilting air desiccation assay

Response to air desiccation was evaluated in the R5 stage. Three whole leaves *per* genotype (n = 6) were collected and exposed to air desiccation at 32 °C. After 0, 6, 24, 36 and 48 h of air exposure, plants were photographed with a Canon Power Shot SX520 HS (14 Mpx), and the wilting rate was assessed.

#### Comparative analysis of genotypes responses to water deficit in V3 and R5

The responses of MUNASQA and TJ2049 to water deficit, applied in V3 and R5 stages, were compared by measuring morphophysiological and biochemical parameters grouped by biological processes (BP) (Table 6). Four treatments were defined: (i) Control-V3, (ii) Control-R5, (iii) Stress-V3 and (iv) Stress-R5, and three sampling times were performed (0, 4 and 8 d of water deficit). Ten plants *per* genotype, treatment and time were collected and used for markers evaluation (n = 240). Additionally, for treatments (i), (iii) and (iv), 50 plants *per* genotype were harvested at physiological maturity (n = 300) to quantify relative yield and calculate the relative yield DSI (Drought Susceptibility Index) according to Fischer and Maurer^61^.

**Table 6:** Markers evaluated in MUNASQA and TJ2049, clustered by biological processes (BP).

Set	Marker
I. Stress response	1. Superoxide dismutase (SOD)
	2. Ascorbate peroxidase (APX)
	3. Phenol peroxidase (POX)
	4. Catalase (CAT)
	5. Free proline (PRO)
	6. Malondialdehyde (MDA)
	7. Total chlorophyll (CHL)
	8. Total carotenoid (CAR)
II. Growth	9. Leaf area index (LAI)
	10. Leaf area ratio (LAR)
	11. Net assimilation rate (NAR)
	12. Relative growth rate (RGR)
	13. Crop growth rate (CGR)
III. Water use	14. Relative water content (RWC)
	15. Water use efficiency (WUE)
	16. Canopy temperature depression (CTD)
	17. Leaf thickness (LT)
	18. Trichome density in abaxial surface (TD_AB)
	19. Trichome density in adaxial surface (TD_AD)
	20. Stomatal density in abaxial surface (SD_AB)
	21. Stomatal density in adaxial surface (SD_AD)
	22. Stomatal aperture

The markers evaluated and their methodologies are detailed below.

#### Markers

As stress response markers, the activities of SOD, APX, POX and CAT proteins were assessed, together with the accumulation of free proline (PRO)^62^, malondialdehyde (MDA)^63^, total chlorophylls (CHL)^64^, and carotenoids (CAR)^65^.

As growth indicators, the plant total leaf area (TLA) and biomass (plant total dry weight) were determined. Then leaf area index (LAI) and leaf area ratio (LAR)^66^, the net assimilation rate (NAR)^67^, relative growth rate (RGR)^68^ and crop growth rate (CGR)^69^ were calculated.

Finally, as water-use parameters, plant RWC and WUE^70^ were calculated. Moreover, the canopy temperature was monitored and recorded using a FLIR ONE-3 thermal camera (0.3456 Mpx) to calculate canopy temperature depression (CTD)^71^.

### Univariate analysis

Data from stomatal apertures were subjected to a two-way ANOVA (Factor 1: genotype, Factor 2: treatment). The remaining data were analyzed through ANOVA with post hoc contrast by Tukey's HSD test. Data were analyzed with InfoStat statistical package^52^ and presented as the arithmetic mean ± SE. Means were considered significantly different at *P* < 0.05.

### Correlations, multivariate analysis and markers selection

The 22 markers strength of association between phenological stages, V3 and R5, was measured by Pearson's correlation analysis adjusted by Bonferroni (*P* < 0.05 indicated as *; *P* < 0.01 ** and *P* < 0.001 ***). Then, the markers correlation with relative yield was assessed. Correlation coefficients (*r*^2^) were classified as “Strong” (> ± 0.60) and “Weak” (below ± 0.59).

All markers were submitted to a PCA to discriminate main associations between markers, genotypes and treatments. However, in PCAs, an increase in the number of comparable variables will reduce the proportion of variance among treatments explained by those variables. Therefore, all markers were grouped by biological processes in (i) “stress response”, (ii) “growth”, and iii) “water use” sets and subjected to independent PCAs to discriminate which markers better explain the variability between genotypes/treatments.

Additionally, the markers were ranked by CF and SW. The markers CF, in terms of their complexity and evaluation cost, was assigned according to 4 categories: easy and cheap (1), easy and expensive (2), complicated and cheap (3) or complicated and expensive (4). Meanwhile, the SW was obtained from PCA variables coefficients (autovectors e1 and e2) that were ranked and classified in “Low” (Low = [− 2, 2]) and High (High = ℝ − [− 2, 2]), according to their weigh on PC1 and PC2. Markers strongly correlated between phenological stages, if possible, with yield, together with CF values of 1 or 2 and “High” SW in both PC, were selected as the most efficient phenotyping markers.

### General guidelines statement

The authors declare that there is no conflict for the use of commercial soybean varieties for scientific research purposes cited in this article in accordance with Argentine law (Law of Seeds and Phytogenetic Creations No. 20,247/73).

## Supplementary Information

Supplementary Figure S1.

Supplementary Figure S2.

Supplementary Figure S3.

Supplementary Table 1.

Supplementary Table 2.

Supplementary Table 3.

Supplementary Table 4.

Supplementary Table 5.

Supplementary Table 6.

Dataset S1.

Dataset S2.

Dataset S3.

Dataset S4.
