# Supplementary material for: Selecting putative drought-tolerance markers in two contrasting soybeans
Source: Sci Rep. 2022 Jun 27;12:10872. doi: 10.1038/s41598-022-14334-3 (PMC9237119; doi:10.1038/s41598-022-14334-3)

**Fig** **S1**. Irrigation treatments and sample times. Three independent assays in V3 and three in R5 stages were carried out. The assays were performed as follows: at 2016/17 season T1 and T2 were used for DSI calculations, and T2 was used for transcriptome sequencing analysis and biochemical measurements, leaf thickness, stomatal and trichome densities. At the 2017-2018 season, stomatal apertures (T3), wilting air desiccation (T4) and a wide range of morphophysiological and biochemical evaluations were evaluated. At the 2018-2019 season, independent replicates of stomatal apertures measurements, wilting air desiccation and morphophysiological and biochemical evaluations assays were repeated (T5 and T6).


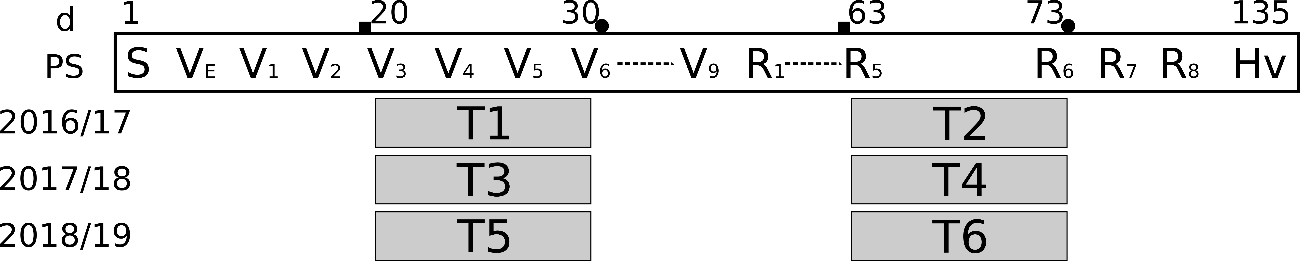

Supplement: Supplementary file 1 — Supplementary Figure S1. [file 41598_2022_14334_MOESM1_ESM.docx]
