# Supplementary material for: Selecting putative drought-tolerance markers in two contrasting soybeans
Source: Sci Rep. 2022 Jun 27;12:10872. doi: 10.1038/s41598-022-14334-3 (PMC9237119; doi:10.1038/s41598-022-14334-3)
Supplement: Supplementary file 2 — Supplementary Figure S2. [file 41598_2022_14334_MOESM2_ESM.docx]

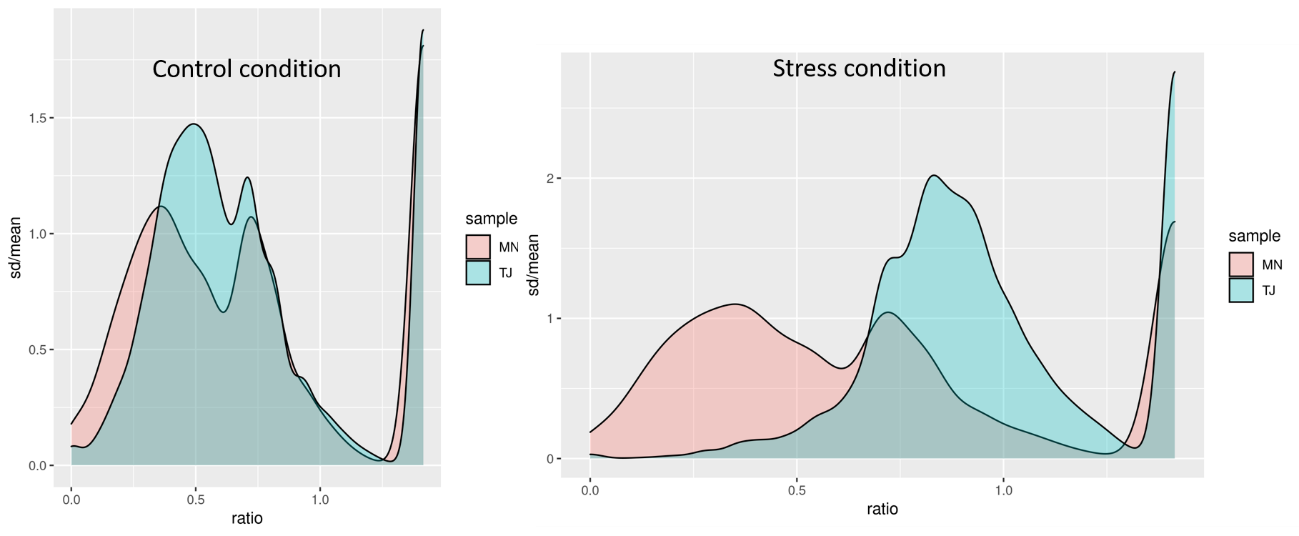


**Fig.S2** Variability among replicates for MN and TJ measured as SD/mean ratio. SD/mean was calculated for each gene for all replicates both in MN and TJ and represented in this graph. The distribution of SD/mean of TJ is much more closely shifted to the right under the stress conditions (One Tiled wilcoxon rank sum test p value < 2.2.10^-16^). The variability among replicates for each transcript (in terms of their respective means) is much wider in TJ stress than in MN stress condition. This excess of variability among replicates (stress) is penalized in the p value of the DEGs, since the differences observed among means are more likely to be due to variability among replicates than between treatments.
