# Supplementary material for: Selecting putative drought-tolerance markers in two contrasting soybeans
Source: Sci Rep. 2022 Jun 27;12:10872. doi: 10.1038/s41598-022-14334-3 (PMC9237119; doi:10.1038/s41598-022-14334-3)
Supplement: Supplementary file 3 — Supplementary Figure S3. [file 41598_2022_14334_MOESM3_ESM.docx]

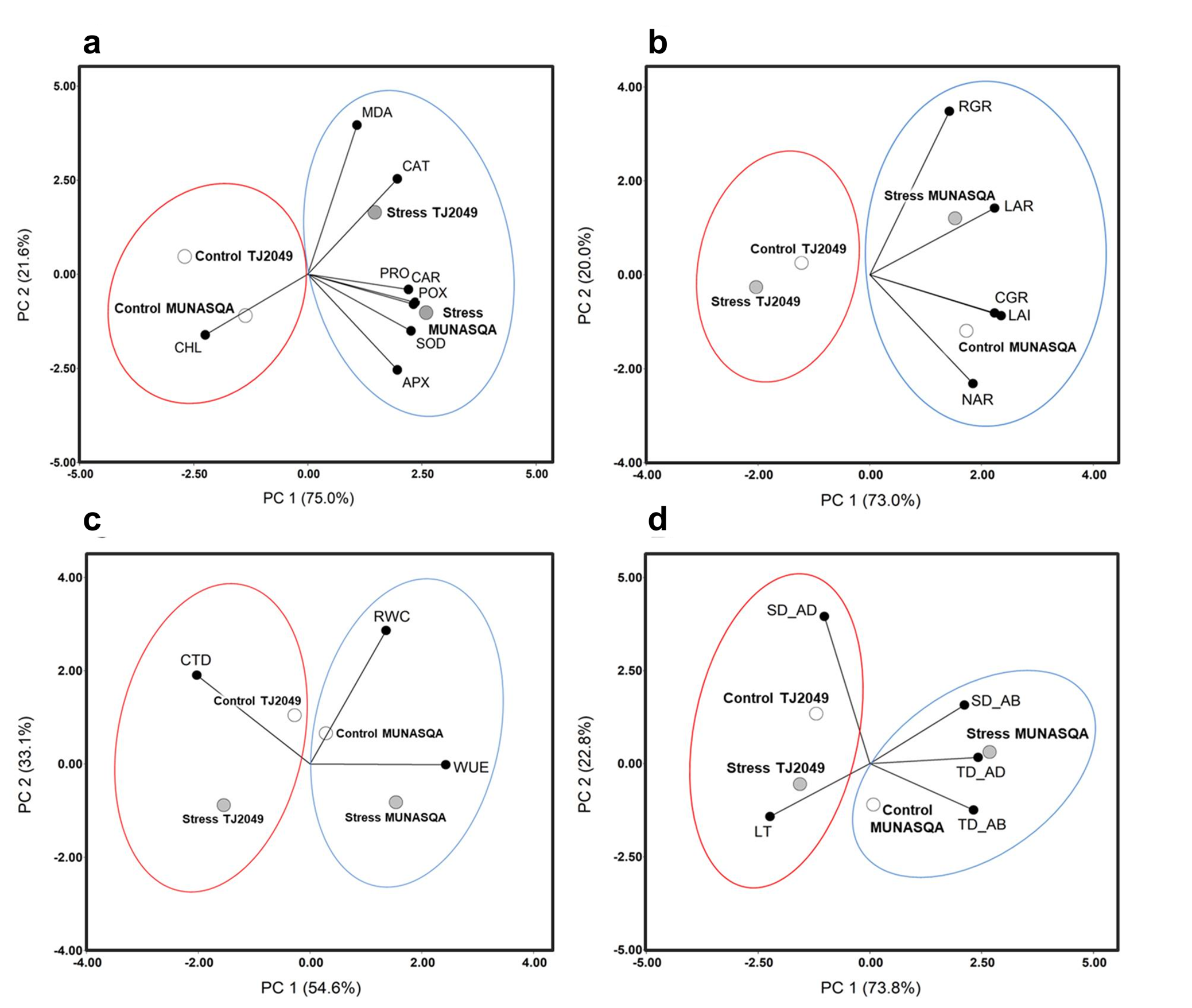


**Fig.S3** PCA for determining markers interaction with genotypes and treatments. Markers Set I: stress-response. PCA was performed using the SOD, APX, POX, CAT, MDA, PRO, CHL and CAR data (**a**). Markers Set II: growth. PCA was performed using LAI, LAR, NAR, RGR and CGR data (**b**). Physiological markers of Set III: water use. PCA was performed using the RWC, WUE and CTD data (**c**). Morphological markers of Set III: water use. PCA was performed using the LT, TD_AB, TD_AD, ST_AB and SD_AD data (**d**). For (**a** to **c**), MUNASQA and TJ2049 were exposed to water deficit (Ψs=-0.65 MPa) and well-watered (Ψs=-0.05 MPa) treatments applied in V3 and R5. Markers were determined at 0, 4 and 8 d after stress treatment was initiated. For (**d**), both genotypes were exposed to the same water regimen in R5. Parameters were determined 21 d after stress imposition.
