# Supplementary material for: Selecting putative drought-tolerance markers in two contrasting soybeans
Source: Sci Rep. 2022 Jun 27;12:10872. doi: 10.1038/s41598-022-14334-3 (PMC9237119; doi:10.1038/s41598-022-14334-3)
Supplement: Supplementary file 4 — Supplementary Table 1. [file 41598_2022_14334_MOESM4_ESM.docx]

**Suppl. Table 1**

Total read counts for treatments, genotypes and biological replicates.

| **Genotype** | **Treatment** | **Rep 1 read counts** | **Rep 2 read counts** | **Rep 3 read counts** | **Total counts** |
| --- | --- | --- | --- | --- | --- |
| **MUNASQA** | Control | 408.185 | 451.706 | 580.699 | 1.440.590 |
|  | Drought | 486.962 | 545.307 | 3.027.343 | 4.059.612 |
| **TJ2049** | Control | 717.287 | 2.491.106 | 1.155.246 | 4.363.639 |
|  | Drought | 460.358 | 1.037.199 | 3.238.357 | 4.735.914 |
