# Supplementary material for: Selecting putative drought-tolerance markers in two contrasting soybeans
Source: Sci Rep. 2022 Jun 27;12:10872. doi: 10.1038/s41598-022-14334-3 (PMC9237119; doi:10.1038/s41598-022-14334-3)
Supplement: Supplementary file 5 — Supplementary Table 2. [file 41598_2022_14334_MOESM5_ESM.docx]

**Suppl. Table 2**

Some DEGs in response to drought that are only present in MUNASQA plants.

| **Gene** | **Log_2_ ratio** | **Annotation** |
| --- | --- | --- |
| **Auxin related proteins** |  |  |
| Glyma.04G252300 | 1,44 | Auxin transporter-like protein 2 |
| Glyma.06G164900 | -1,9 | Auxin response factor (ARF) |
| Glyma.09G011200 | 1,55 | Auxin-induced in root cultures protein |
| **Ethylene-responsive proteins** |  |  |
| Glyma.15G152000 | -2 | Ethylene-responsive element binding protein |
| **Helix-loop-helix proteins** |  |  |
| Glyma.04G199900 | 4,19 | Hélix-loop-helix protein BHLH137 |
| **Zinc finger proteins** |  |  |
| Glyma.01G236800 | 1,81 | Zing finger protein |
| Glyma.12G196200 | -2,33 | Zing finger protein |
| Glyma.15G268100 | -2,05 | Zing finger protein |
| Glyma.20G149700 | -1,68 | Zing finger protein |
| Glyma.13G009100 | -3,26 | Zing finger protein |
| Glyma.14G129100 | 2,64 | C3H4 type zinc finger protein |
| Glyma.11G243400 | -2,37 | Ring/U-box domain-containing protein (XERICO) |
| **WRKY transcription factor family** |  |  |
| Glyma.05G160800 | 2,1 | WRKY transcription factor 65-related |
| Glyma.06G077400 | 1,52 | WRKY transcription factor 11 |
| **NAC family** |  |  |
| Glyma.06G248900 | -2,45 | NAC domain related protein |
| Glyma.07G229100 | -3,71 | NAC domain related protein |
| **Protein kinase family** |  |  |
| Glyma.14G118200 | -3,79 | Leucine-rich repeat protein |
| Glyma.06G062100 | -5,3 | CBL-interacting serine/threonine protein kinase 25 |
| **MPKs** |  |  |
| Glyma.09G256000 | 1,78 | Mitogen-activated protein kinase |
| **Response to ABA** |  |  |
| Glyma.04G180400 | 1,98 | Dehydratation-responsive protein RD22 |
| Glyma.14G140900 | -4,99 | Dehydratation-responsive protein RD22 |
| Glyma.01G060300 | -2,74 | NINJA_B |
| **Dof family** |  |  |
| Glyma.07G198900 | 3,3 | Dof family protein |
| **CDPKs** |  |  |
| Glyma.11G077300 | 1,84 | Calcium-dependent protein kinase |
| **Cytochrome P450s** |  |  |
| Glyma.09G049100 | 1,24 | Cytochrome P450 81D1 related enzyme |
| **MADS BOX proteins** |  |  |
| Glyma.17G081200 | -1,84 | MADS BOX protein |
| Glyma.03G083900 | -2,75 | MADS BOX protein |
| **Pathogen related proteins** |  |  |
| Glyma.15G218900 | 1,29 | Pahotogenesis-related protein Bet v1 |
| Glyma.08G230500 | 1,23 | Pahotogenesis-related protein Bet v1 |
| **ATP binding proteins** |  |  |
| Glyma.11G093900 | -1,89 | ATP binding cassette transporter |
