# Supplementary material for: Selecting putative drought-tolerance markers in two contrasting soybeans
Source: Sci Rep. 2022 Jun 27;12:10872. doi: 10.1038/s41598-022-14334-3 (PMC9237119; doi:10.1038/s41598-022-14334-3)
Supplement: Supplementary file 6 — Supplementary Table 3. [file 41598_2022_14334_MOESM6_ESM.docx]

**Suppl. Table 3**

Antioxidant isoforms coding genes identified in MACE RNA-Seq analysis.

| **Gene ID** | **Gene function** | **MUNASQA**  **(Stress vs Control)** | **TJ2049**  **(Stress vs Control)** |
| --- | --- | --- | --- |
| *Glyma.19g42890* | SOD1 | ↑ | ─ |
| *Glyma.20g33880* | SOD2 | ↑ | ─ |
| *Glyma.11g15680* | APX1 | ↓ | ↑ |
| *Glyma.12g07780* | APX2 | ↓ | ↑ |
| *Glyma.12g03610* | APX5 | ↓ | ↓ |
| *Glyma.06g02040* | CAT1 | ↓ | ─ |
| *Glyma.17g38140* | CAT2 | ↓ | ↑ |
| *Glyma.14g39810* | CAT3 | ↓ | ─ |
| *Glyma.04g01920* | CAT4 | ↑ | ↓ |
| *Glyma.17g01720* | PRX2 | ↑ | ↑ |
| *Glyma.05g22180* | PRX35 | ─ | ↑ |
| *Glyma.11g02620* | GPX8 | ↑ | ─ |
| *Glyma.18g49031* | TRX-Ct. | ↑ | ─ |
| *Glyma.04g02330* | TRX-Ct. | ↓ | ↓ |
| *Glyma.06g02380* | TRX-Ct. | ↓ | ↓ |
| *Glyma.17g05150* | TRX-Chl. | ↑ | ↑ |
| *Glyma.01g42840* | TRX-Chl | ↓ | ↑ |

***SOD1***: chloroplastic copper-zinc superoxide dismutase; ***SOD2***: chloroplastic iron-manganese superoxide dismutase; ***APX1, 2***: cytosolic ascorbate peroxidase; ***APX5***: peroxisomal ascorbate peroxidase; ***CAT1***: peroxisomal and mitochondrial catalase; ***CAT2, 3, 4***: peroxisomal catalase; ***PRX2***: cell wall peroxiredoxin; ***PRX35***: extracellular peroxiredoxin; ***GPX8*:** nuclear and cytosolic glutathione peroxidase; ***TRX-Ct*:** cytosolic thioredoxin; ***TRX-Chl*:** chloroplastic thioredoxin. Symbols: **↑** gene upregulation; ↓ gene downregulation; ─ unaltered gene expression.
