# Supplementary material for: Selecting putative drought-tolerance markers in two contrasting soybeans
Source: Sci Rep. 2022 Jun 27;12:10872. doi: 10.1038/s41598-022-14334-3 (PMC9237119; doi:10.1038/s41598-022-14334-3)
Supplement: Supplementary file 7 — Supplementary Table 4. [file 41598_2022_14334_MOESM7_ESM.docx]

**Suppl. Table 4**

Effect of mild water deficit on stress-response enzymatic markers measured in MUNASQA and TJ2049 soybean genotypes. Values of Superoxide dismutase (SOD), Ascorbate peroxidase (APX), Phenol peroxidase (POX) and Catalase (CAT) enzymatic activities were obtained from plants submitted to water deficit (Ψs=-0.65 MPa) and well-watered treatments (Ψs=-0.05 MPa) applied in R_5_ phenological stage. The experiment was conducted with both genotypes (n= 5 per genotype/treatment), which were evaluated 72 hs after stress imposition. Average values followed by the same uppercase letter in the column do not differ statistically, according to Tukey’s HSD test at 5%.

| **Genotype and Treatment** | **SOD**  **(µmol O_2_^-^ gDW^-1^ min^-1^)** | | **APX**  **(µmol Asa gDW^-1^ min^-1^)** | | **POX**  **(µmol Purpurogalline gDW^-1^ min^-1^)** | | **CAT**  **(µmol H_2_O_2_ gDW^-1^ min^-1^)** | |
| --- | --- | --- | --- | --- | --- | --- | --- | --- |
| **TJ2049 Control** | 70.57 | **A** | 42.13 | **A** | 141.5 | **B** | 103.70 | **B** |
| **TJ2049 Stress** | 72.67 | **A** | 90.50 | **B** | 121.33 | **A** | 138.83 | **C** |
| **MUNASQA Control** | 118.29 | **B** | 151.47 | **C** | 146.10 | **C** | 93.07 | **A** |
| **MUNASQA Stress** | 199.78 | **C** | 160.13 | **D** | 151.03 | **D** | 104.07 | **B** |
| **Standard Error** | 1.50 | | 1.22 | | 0.69 | | 1.87 | |
