# Supplementary material for: Selecting putative drought-tolerance markers in two contrasting soybeans
Source: Sci Rep. 2022 Jun 27;12:10872. doi: 10.1038/s41598-022-14334-3 (PMC9237119; doi:10.1038/s41598-022-14334-3)
Supplement: Supplementary file 8 — Supplementary Table 5. [file 41598_2022_14334_MOESM8_ESM.docx]

**Suppl. Table 5**

Meteorological variables measured during the six experiments performed in greenhouse conditions.

| **Experiments** | **Day length (hours)** | **ST (°C ± 2)** | **ET (°C ± 2)** | **RH (%)** | **PAR (μmol m^-2^s^-1^)** |
| --- | --- | --- | --- | --- | --- |
| 1 | 12 | 25 | 30 | 94 | 550 |
| 2 | 12 | 24 | 28 | 90 | 534 |
| 3 | 12 | 24 | 29 | 92 | 420 |
| 4 | 12 | 26 | 30 | 87 | 500 |
| 5 | 12 | 23 | 29 | 85 | 597 |
| 6 | 12 | 25 | 31 | 90 | 530 |

Mean values of day length, substrate temperature (ST), environmental temperature (ET), relative humidity (RH) and incident solar radiation (PAR), recorded and averaged in six phenotyping trials.
