# Supplementary material for: Selecting putative drought-tolerance markers in two contrasting soybeans
Source: Sci Rep. 2022 Jun 27;12:10872. doi: 10.1038/s41598-022-14334-3 (PMC9237119; doi:10.1038/s41598-022-14334-3)
Supplement: Supplementary file 9 — Supplementary Table 6. [file 41598_2022_14334_MOESM9_ESM.docx]

**Suppl. Table 6**

Primer sequences used in MACE validation by qRT-PCR.

| **Gene** | **Gene function** | **Primers sequences (5’3’)** |
| --- | --- | --- |
| *Glyma.12G051100* | F-BOX | **F:** AGATAGGGAAATGGTGCAGGT |
|  |  | **R:** CTAATGGCAATTGCAGCTCTC |
| *Glyma.06g143300* | EXPANSIN A-8 | **F:** CCATTGTAGCAACCTCTCTTCT |
|  |  | **R:** ATGCATCACCTCCACCATAAA |
| *Glyma.04g199900* | BHLH 137 | **F:** TCTAGACACCCTCTTGGTTAGA |
|  |  | **R:** AGTAGTGGCTTGGTTGTTAGG |
| *Glyma.08g311200* | Citrate synthase | **F:** GGGCTGTTGGAGCTCTATATG |
|  |  | **R:** CCCTCAATGAATGCTGGAATG |
| *Glyma.04g010300* | CAMP-responsive element | **F:** ATCTCGAATGAGGAAGCAGAAG |
|  |  | **R:** GAGGTTCACCGATGTGAGAAT |
| *Glyma.05g103600* | Peroxidase 35 | **F:** CCACCCTTCGCCTATTCTTT |
|  |  | **R:** TCCTTCTCTGCCTGGTTATTTC |
| *Glyma.04g202300* | Mitochondrial outer membrane protein 25 | **F:** CCCCATCTTCATTAGCTCCAG |
|  |  | **R:** TTGGATGAAGTAAGAGGTTAAGGG |
| *Glyma.20g167500* | ABA-WDS protein | **F:** GTGGCTATGAAGGTGGTTACA |
|  |  | **R:** GCCACCAGAAGTCTCATCAA |
| *Glyma.09g185500* | Dehydrin | **F:** CGCACACTGAAGCAGGATATAG |
|  |  | **R:** CTCCAGGAAGCTTCTCCTTAATC |
| *Glyma.13g009100* | RHA1 | **F:** TTCGTCGTTGTCTCCTTCAC |
|  |  | **R:** GTGTCCTGCTTCTGAACTCTT |
| *Glyma.13g278000* | Defensin like protein | **F:** GTGCTTCCTCTTCCTTGTTCT |
|  |  | **R:** CTGTATGTATCAGCCAGGTTCTC |
